# Optimization of the IPP Precursor Supply for the Production of Lycopene, Decaprenoxanthin and Astaxanthin by *Corynebacterium glutamicum*

**DOI:** 10.3389/fbioe.2014.00028

**Published:** 2014-08-20

**Authors:** Sabine A. E. Heider, Natalie Wolf, Arne Hofemeier, Petra Peters-Wendisch, Volker F. Wendisch

**Affiliations:** ^1^Faculty of Biology and Center for Biotechnology (CeBiTec), Bielefeld University, Bielefeld, Germany

**Keywords:** carotenoid production, genome-reduced *Corynebacterium glutamicum*, MEP pathway, synthetic operons, astaxanthin

## Abstract

The biotechnologically relevant bacterium *Corynebacterium glutamicum*, currently used for the million ton-scale production of amino acids for the food and feed industries, is pigmented due to synthesis of the rare cyclic C50 carotenoid decaprenoxanthin and its glucosides. The precursors of carotenoid biosynthesis, isopenthenyl pyrophosphate (IPP) and its isomer dimethylallyl pyrophosphate, are synthesized in this organism via the methylerythritol phosphate (MEP) or non-mevalonate pathway. Terminal pathway engineering in recombinant *C. glutamicum* permitted the production of various non-native C50 and C40 carotenoids. Here, the role of engineering isoprenoid precursor supply for lycopene production by *C. glutamicum* was characterized. Overexpression of *dxs* encoding the enzyme that catalyzes the first committed step of the MEP-pathway by chromosomal promoter exchange in a prophage-cured, genome-reduced *C. glutamicum* strain improved lycopene formation. Similarly, an increased IPP supply was achieved by chromosomal integration of two artificial operons comprising MEP pathway genes under the control of a constitutive promoter. Combined overexpression of *dxs* and the other six MEP pathways genes in *C. glutamicum* strain LYC3-MEP was not synergistic with respect to improving lycopene accumulation. Based on *C. glutamicum* strain LYC3-MEP, astaxanthin could be produced in the milligrams per gram cell dry weight range when the endogenous genes *crtE*, *crtB*, and *crtI* for conversion of geranylgeranyl pyrophosphate to lycopene were coexpressed with the genes for lycopene cyclase and β-carotene hydroxylase from *Pantoea ananatis* and carotene C(4) oxygenase from *Brevundimonas aurantiaca*.

## Introduction

Carotenoids are ubiquitous natural pigments with colors ranging from yellow to red. They are composed of isoprene units and belong to the family of terpenoids. These pigments do not only play important and versatile roles in their biological hosts, but are also suggested to have a beneficial effect on human health. Furthermore, they are intensively applied for food and beverage coloration (Downham and Collins, 2000; Gassel et al., 2013). Hence, carotenoids have received extensive considerable attention and especially the interest for an efficient and environmental-friendly production by microbial hosts is increasing (Lee and Schmidt-Dannert, 2002; Das et al., 2007; Harada and Misawa, 2009; Cutzu et al., 2013). In order to compete with already existing production processes, such as chemical synthesis or extraction from organic material, the large-scale production in microbial hosts requires process as well as strain optimization. One of the most common strategies for enhanced production is the efficient supply of precursor molecules as all carotenoids derive from the universal C5 precursor molecule IPP and its isomer DMAPP. IPP and DMAPP can be synthesized via two independent pathways, the mevalonate (MVA) and the 2-methylerythritol 4-phosphate (MEP) pathway (Rodriguez-Concepcion and Boronat, 2002). The MVA pathway starts from acetyl-CoA and operates mainly in eukaryotes (mammals, fungi, in the cytoplasm of plant cells), archaea, and a limited number of bacteria. The MEP pathway that starts from pyruvate and glyceraldehyde 3-phosphate and proceeds via the eponymous intermediate MEP was identified much later (Rohmer et al., 1993) and is found in most bacteria as well as in plant plastids (Rohmer, 1999; Lange et al., 2000; Lee and Schmidt-Dannert, 2002). Both pathways also differ regarding redox and energy requirements (Steinbüchel, 2003). As the MEP pathway is present in several pathogens such as *Plasmodium falciparum* and *Mycobacterium tuberculosis*, but not in mammals, it is considered a drug target (Jomaa et al., 1999; Testa and Brown, 2003).

**Figure 1:**
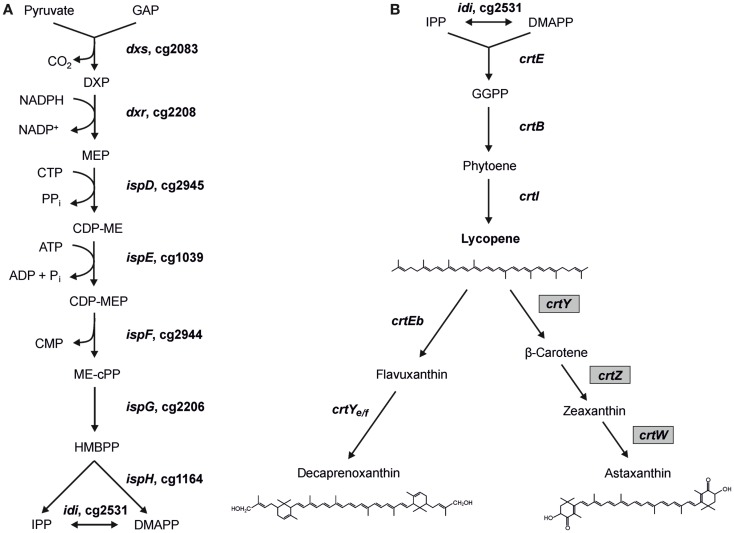
**Scheme of the MEP pathway (A) and of decaprenoxanthin biosynthesis in *C. glutamicum* (B) with heterologous astaxanthin biosynthesis**. Gene names from *C. glutamicum* (and gene IDs for MEP pathway genes) as well as gene names from *Pantoea ananatis* and *Brevundimonas aurantiaca* (gray boxes) are indicated. The structures of the endogenous C50 carotenoid decaprenoxanthin and the heterologous C40 carotenoid astaxanthin are given (GAP, glyceraldehyde 3-phosphate; DXP, 1-deoxy-d-xylulose 5-phosphate; MEP, 2-methylerythritol 4-phosphate; CDP-ME, 4-diphosphocytidyl-2-methylerythritol; CDP-MEP, 4-diphosphocytidyl-2-methylerythritol 2-phosphate; ME-cPP, 2-methylerythritol 2,4-cyclopyrophosphate; HMBPP, 4-hydroxy-3-methyl-but-2-enyl pyrophosphate; IPP, isopentenyl pyrophosphate; DMAPP, dimethylallyl pyrophosphate).

The MEP pathway consists of nine reactions catalyzed by eight enzymes (Figure 1) starting with the transfer of an acetaldehyde group derived from pyruvate to GAP, forming 1-deoxy-d-xylulose 5-phosphate (DXP), in the reaction of DXP synthase Dxs (EC 2.2.1.7). The intermediate DXP is also the precursor for thiamine (vitamine B_1_) (Begley et al., 1999) and pyridoxol (vitamine B_6_) (Hill et al., 1996) biosynthesis. Subsequently, DXP reductoisomerase Dxr (EC 1.1.1.267) converts DXP to MEP using NADPH as cofactor. MEP is then converted to the cyclic diphosphate 2C-methyl-d-erythritol-2,4-cyclodiphosphate (ME-cPP) by the three enzymes IspD, IspE, and IspF (Gräwert et al., 2011). ME-cPP is then converted to IPP and DMAPP by a reduction and elimination reaction catalyzed by the two iron–sulfur proteins IspG and IspH (Rohdich et al., 2004). It is proposed that flavodoxin is an essential redox partner for one of the enzymes (Adam et al., 2002; Gräwert et al., 2004; Puan et al., 2005). IPP and DMAPP can be synthesized independently by IspH (Gräwert et al., 2004). IPP and DMAPP often do not occur in the same ratio as for example in *Escherichia coli* IPP is synthesized in a 5:1 proportion to DMAPP (Rohdich et al., 2002; Gräwert et al., 2004; Xiao et al., 2008). The IPP:DMAPP isomerase Idi (EC 5.3.3.2) facilitates the isomerization between IPP and DMAPP. In the case of microorganisms using the MVA pathway produce/synthesize IPP exclusively, isomerases are essential enzymes, whereas in bacteria possessing the MEP pathway *idi* is not essential for the survival of the cells (Hahn et al., 1999; Julsing et al., 2007).

*Corynebacterium glutamicum* is a pigmented Gram-positive bacterium with a long and safe history in the food and feed sector as it is used for the fermentative production of amino acids. Annually, about 2.6 million tons of l-glutamate and about 1.95 million tons of l-lysine are produced biotechnologically worldwide (Ajinomoto, Food Products Business. Available from http://www.ajinomoto.com/en/ir/pdf/Food-Oct2012.pdf and /Feed-useAA-Oct2013.pdf, Cited 18 March 2014). Besides amino acids, the diamines cadaverine and putrescine (Mimitsuka et al., 2007; Schneider and Wendisch, 2010) and the alcohols ethanol and isobutanol (Sakai et al., 2007; Blombach and Eikmanns, 2011), among others, can be produced from sugars by recombinant *C. glutamicum* strains. Furthermore, access of *C. glutamicum* to alternative feed stocks like glycerol from the biodiesel process (Meiswinkel et al., 2013), pentoses from lignocellulosics (Gopinath et al., 2011), amino sugars (Uhde et al., 2013; Matano et al., 2014), starch (Seibold et al., 2006), and β-glucans (Tsuchidate et al., 2011) has been engineered.

Recently, the potential of *C. glutamicum* for production of carotenoids has been explored. *C. glutamicum* synthesizes the cyclic C50 carotenoid decaprenoxanthin and its glucosides (Figure 1). Its carotenogenic pathway and the respective genes have been elucidated (Krubasik et al., 2001; Heider et al., 2012, 2014a) and overproduction of the C50 carotenoids decaprenoxanthin, sarcinaxanthin, and C.p. 450 in the milligrams per gram cell dry weight (DCW) range by *C. glutamicum* was achieved by metabolic engineering of the terminal carotenoid pathway (Heider et al., 2014a). Moreover, the heterologous production of the C40 carotenoids β-carotene and zeaxanthin could be established (Heider et al., 2014a) and hydroxylated carotenoids could be produced either as aglycons or as di-glucosides (Heider et al., 2014a). Engineering of *C. glutamicum* for the production of a sesquiterpene, (+)-valencene, was possible as well (Frohwitter et al., 2014).

Based on its genome sequence, all genes of the MEP pathway of *C. glutamicum* have been putatively assigned. However, neither have the respective genes or enzymes of the MEP pathway been functionally analyzed nor has engineering for an increased IPP supply been reported. The MEP pathway genes are distributed over the genome of *C. glutamicum*. The MEP pathway genes *dxs* (cg2083), *ispH* (cg1164), and *idi* (cg2531) are monocistronic, while *dxr* (cg2208), *ispD* (cg2945), *ispE* (cg1039), *ispF* (cg2944), and *ispG* (cg2206) belong to operons. *IspE* is the third gene of the operon cg1037-*ksgA*-*ispE*-cg1040-*pdxK* with genes for a putative resuscitation-promoting factor (cg1037), putative dimethyladenosine transferase KsgA, and putative pyridoxamine kinase PdxK. *IspD* and *ispF* are encoded in the cg2946-*ispDF* operon with cg2946, which codes for a CarD-like transcriptional regulator. *Dxr* and *ispG* are organized in a transcriptional unit separated by an uncharacterized gene (cg2207) putatively encoding a membrane-embedded Zn-dependent protease. In bacteria, two bottlenecks in the MEP pathway were proposed. On the one hand, DXP synthase, which catalyzes the first reaction is claimed to be rate-limiting (Sprenger et al., 1997; Xiang et al., 2007) and is essential in *E. coli* (Sauret-Gueto et al., 2003) and *Bacillus subtilis* (Julsing et al., 2007) and possibly further bacteria. On the other hand, overproduction of Idi, which is not essential in bacteria possessing the MEP pathway (Hahn et al., 1999; Julsing et al., 2007), improved carotenoid production (Harker and Bramley, 1999; Kim and Keasling, 2001).

In this study, two synthetic operons (*ispDFE* and *dxr-ispGH*) under control of the strong promoter P*_tuf_* of the *C. glutamicum* translation elongation factor EF-Tu gene were integrated into the prophage-cured, genome-reduced *C. glutamicum* strain MB001 (Baumgart et al., 2013). Furthermore, *dxs* was overexpressed from the chromosome by exchanging the endogenous promoter with the P*_tuf_* promoter. Finally, *idi* was overexpressed from an IPTG-inducible plasmid. The genome-reduced strain overexpressing all of the eight MEP pathway genes was then shown to be suitable for production of lycopene and endogenous decaprenoxanthin as well as for production of the non-native astaxanthin.

## Materials and Methods

### Bacterial strains, media and growth conditions

The strains and plasmids used in this work are listed in Table 1. *C. glutamicum* ATCC13032 was used as wild type (WT), for metabolic engineering the prophage-cured *C. glutamicum* MB001 (Baumgart et al., 2013) was used as platform strain. Precultivation of *C. glutamicum* strains was performed in LB medium or LB with glucose. For cultivation in CGXII medium (Eggeling and Reyes, 2005), precultivated cells were washed once with CGXII medium without carbon source and inoculated to an initial OD_600_ of 1. Glucose was added as carbon and energy source to a concentration of 100 mM. Standard cultivations of *C. glutamicum* were performed at 30°C in a volume of 50 ml in 500 ml flasks with two baffles shaking at 120 rpm. The OD_600_ was measured in dilutions using a Shimadzu UV-1202 spectrophotometer (Duisburg, Germany). Alternatively, cultivations were performed in 1 ml volume in microtiterplates at 1100 rpm at 30°C using Biolector^®^ micro fermentation system (m2p-labs GmbH, Baesweiler, Germany). For cloning, *E. coli* DH5α was used as host and cultivated in LB medium at 37°C. When appropriate, kanamycin or spectinomycin was added to concentrations of 25 and 100 μg ml^−1^, respectively. Gene expression was induced by adding 50 μM and 1 mM IPTG, respectively, at inoculation of the main culture.

**Table 1:** **Strains and plasmids used in this study**.

Strain, plasmid	Relevant characteristics	Source or reference
***C. glutamicum* STRAINS**		
WT	ATCC 13032	Abe et al. (1967)
MB001	Prophage-cured ATCC 13032; in-frame deletion of prophages cgp1 (cg1507-cg1524), cgp2 (cg1746-cg1752), and cgp3 (cg1890-cg2071)	Baumgart et al. (2013)
LYC3	*crtY_e_Y_f_Eb* deletion mutant of *C. glutamicum* MB001	This work
LYC3-P*_tuf_dxs*	LYC3 derivative with *dxs* (cg2083) under control of the P_tuf_ promoter integrated into the intergenic region of cg2083 and cg2084	This work
LYC3-Op1	LYC3 derivative with *ispD* (cg2945), *ispF* (cg2944), and *ispE* (cg1039) under control of the P_tuf_ promoter integrated into the cgp2 cured region between cg1745 and cg1753	This work
LYC3-Op2	LYC3 derivative with *dxr* (cg2208), *ispG* (cg2206), and *ispH* (cg1164) under control of the P_tuf_ promoter integrated into the cgp1 cured region between cg1506 and cg1525	This work
LYC3-Op1Op2	LYC3-Op2 derivative with *ispD* (cg2945), *ispF* (cg2944), and *ispE* (cg1039) under control of the P_tuf_ promoter integrated into the cgp2 cured region between cg1745 and cg1753	This work
LYC3-MEP	LYC3-Op1Op2 derivative with with *dxs* (cg2083) under control of the P_tuf_ promoter integrated into the intergenic region of cg2083 and cg2084	This work
**OTHER STRAINS**		
*E. coli* DH5α	F^−^*thi*-1 *endA1 hsdR17*(r^−^m^−^) *supE44* Δ*lacU169* (ϕ80*lacZ*ΔM15) *recA1 gyrA96 relA1*	Hanahan (1983)
*Pantoea ananatis*	ATCC 19321	Misawa et al. (1990)
*Brevundimonas aurantiaca*	ATCC 15266	Abraham et al. (1999)
**PLASMIDS**		
pK19*mobsacB*	Km^R^; *E. coli*/*C. glutamicum* shuttle vector for construction of insertion and deletion mutants in *C. glutamicum* (pK18 *oriV_Ec_ sacB lacZ*α)	Schäfer et al. (1993)
pK19*mobsacB*-Δ*crtYEb*	pK19*mobsacB* with a *crtY_e_Y_f_Eb* deletion construct	Heider et al. (2014a)
pK19*mobsacB*-P*_tuf_dxs*	pK19*mobsacB* derivative with a *tuf* promoter region (200 bp upstream of the coding sequence of the *tuf* gene(cg0587) construct for the promoter exchange of *dxs*	This work
pK19*mobsacB*-Op1	pK19*mobsacB* derivative containing the artificial operon *ispDFE* under the control of the P*_tuf_* promoter with an additional ribosome binding site in front of *ispE* for integration in the cgp2 cured region of *C. glutamicum* MB001	This work
pK19*mobsacB*-Op2	pK19*mobsacB* derivative containing the artificial operon *dxr_ispGH* under the control of the P*_tuf_* promoter with addition ribosome binding sites in front of *ispG* and *ispH* for integration in the cgp2 cured region of *C. glutamicum* MB001	This work
pVWEx1	Km^R^; *E. coli*/*C. glutamicum* shuttle vector for regulated gene expression (P_tac_, *lacI*^q^, pCG1 *oriV_Cg_*)	Peters-Wendisch et al. (2001)
pVWEx1-*crtEBI*	pVWEx1 derivative for IPTG-inducible expression of *crtE* and the cluster *crtBI* from *C. glutamicum* containing artificial ribosome binding sites each	Heider et al. (2014a)
pVWEx1-*dxs*	pVWEx1 derivative for IPTG-inducible overexpression of *dxs* (cg2083) containing an artificial ribosome binding site in front of the gene	This work
pVWEx1-*idi*	pVWEx1 derivative for IPTG-inducible overexpression of *idi* (cg2531) containing an artificial ribosome binding site in front of the gene	This work
pVWEx1-*glpFKD*	pVWEx1 derivative for IPTG-inducible overexpression of *glpF*, *glpK*, and *glpD* from *E. coli* MG1655	Rittmann et al. (2008)
pEKEx3	Spec^R^; *E. coli*/*C. glutamicum* shuttle vector for regulated gene expression (P_tac_, *lacI*^q^, pBL1 *oriV_Cg_*)	Stansen et al. (2005)
pEKEx3-*crtEbY*	pEKEx3 derivative for IPTG-inducible expression of *crtEb* and *crtY* from *C. glutamicum* containing artificial ribosome binding sites in front of each gene	Heider et al. (2014a)
pEKEx3-*crtY*	pEKEx3 derivative for IPTG-inducible expression of *crtY* from *P. ananatis* containing an artificial ribosome binding site in front of the gene	Heider et al. (2014a)
pEKEx3-*crtYZ*	pEKEx3 derivative for IPTG-inducible expression of *crtY* and *crtZ* from *P. ananatis* containing artificial ribosome binding sites in front of each gene	Heider et al. (2014a)
pEKEx3-*crtYZW*	pEKEx3 derivative for IPTG-inducible expression of *crtY* and *crtZ* from *P. ananatis* and *crtW* of *Brevundimonas aurantiaca* containing artificial ribosome binding sites in front of each gene	This work
pEKEx3-*crtZWY*	pEKEx3 derivative for IPTG-inducible expression of *crtY* and *crtZ* from *P. ananatis* and *crtW* of *Brevundimonas aurantiaca* containing artificial ribosome binding sites in front of each gene in the order as depicted by the name	This work
pEKEx3-*dxs*	pEKEx3 derivative for IPTG-inducible overexpression of *dxs* (cg2083) containing an artificial ribosome binding site in front of the gene	This work

### Recombinant DNA work

Plasmids were constructed in *E. coli* DH5α from PCR-generated fragments (KOD, Novagen, Darmstadt, Germany) and isolated with the QIAprep spin miniprep kit (QIAGEN, Hilden, Germany). Oligonucleotides used in this study were obtained from Eurofins MWG Operon (Ebersberg, Germany) and are listed in Table 2. Standard reactions like restriction, ligation, and PCR were performed as described previously (Sambrook and Russell, 2001). Besides the common ligation reaction, the Gibson assembly has been applied for the construction of plasmids (Gibson et al., 2009). If applicable, PCR products were purified using the PCR purification kit or MinElute PCR purification kit (QIAGEN, Hilden, Germany). For transformation of *E. coli*, the RbCl method was used (Hanahan, 1983) and *C. glutamicum* was transformed via electroporation (van der Rest et al., 1999) at 2.5 kV, 200 Ω, and 25 μF. All cloned DNA fragments were shown to be correct by sequencing.

**Table 2:** **Oligonucleotides used in this study**.

Oligonucleotide	Sequence (5′ → 3′)
*crtEb*-A	AAAACCCGGGACTACCACTCCCGAGGTT
*crtEb*-B	*CCCATCCACTAAACTTAAACA*TAGAATTAGTCTTATTTTTTCCATCAT
*crtEb*-C	*TGTTTAAGTTTAGTGGATGGG*ACGATACTGCTAATAGCAATTCATCAGATATAA
*crtEb*-D	AAAACCCGGGATGTGTGGGAGGCTTCGC
*crtEb*-E	GGAGACTCAGCGTTTATGTC
*crtEb*-F	AAAACAATGCGCAGCGCA
*crtY*-A	AAAAGGATCCAGTCGGCTTCAGCATCC
*crtY*-B	*CCCATCCACTAAACTTAAACA*TGAAATATCGATGATAGGGATCAA
*crtY*-E	TTGCACCTGCTGGATACGAA
*crtY*-F	ATCGCTGCTGAAGGAGATGT
1	*GCAGGTCGACTCTAGAGGATCCCC*GTGCTTCGCATCGTCTATGTC
2	*CATTCGCAGGGTAACGGCCA*ATAGTTGGGGGAATTTATAAGGATTTG
3	*CAAATCCTTATAAATTCCCCCAACTAT*TGGCCGTTACCCTGCGAATG
4	*GGGATTCGTGTAGACGACAT*TGTATGTCCTCCTGGACTTC
5	*GAAGTCCAGGAGGACATACA*ATGTCGTCTACACGAATCCC
6	*CGCCTTAGCGGTAATTTTCATCT***GAAGGGCCTCCTTTC**TTAAGCCTTCCACACCACTGC
7	*GCAGTGGTGTGGAAGGCTTAA***GAAAGGAGGCCCTTCAG**ATGAAAATTACCGCTAAGGCG
8	*CTAATGGACGGTGAAGTATCATTTATG*TTATGAAACAGTCAAAATGTGTGC
9	*GCACACATTTTGACTGTTTCATAA*CATAAATGATACTTCACCGTCCATTAG
10	*CCAGTGAATTCGAGCTCGGTACCC*CGCCGTATGTAACAAGATTTG
11	*GCAGGTCGACTCTAGAGGATCCCC*CAGTGAAGGATCGGTGCG
12	*CATTCGCAGGGTAACGGCCA*CCTATCTGCTGGCCGGTG
13	*CACCGGCCAGCAGATAGG*TGGCCGTTACCCTGCGAATG
14	*GATCTTTTTAGTCACGACTCCCAT*TGTATGTCCTCCTGGACTTC
15	*GAAGTCCAGGAGGACATACA*ATGGGAGTCGTGACTAAAAAGATC
16	*CTAGAAAAGGAAGCCGCAT***CTGAAGGGCCTCCTTTC**TTACAAGTTGGTTGCCAACCG
17	*CGGTTGGCAACCAACTTGTAA***GAAAGGAGGCCCTTCAG**ATGCGGCTTCCTTTTCTAG
18	*GCTGATAACAGGTGAGCTCAT***CTGAAGGGCCTCCTTTC**TTACTTGGTTACCTTCACTTCAG
19	*CTGAAGTGAAGGTAACCAAGTAA***GAAAGGAGGCCCTTCAG**ATGAGCTCACCTGTTATCAGC
20	*TCTTACTACTTGCGCTAGGTACAG*TTAATTCTTGTGGCGCAGC
21	*GCTGCGCCACAAGAATTAA*CTGTACCTAGCGCAAGTAGTAAGA
22	*CCAGTGAATTCGAGCTCGGTACCC*CTGCTCATCCTTCAACAACGT
23	TGGCCGTTACCCTGCGAATG
24	TGTATGTCCTCCTGGACTTC
25	*GAAGTCCAGGAGGACATACA*ATGGGAATTCTGAACAGTATTTC
26	*CCAGTGAATTCGAGCTCGGTACCC*CACACTATGCGTGGTATCG
27	*GCAGGTCGACTCTAGAGGATCCCC*CTGTCACTTTCCACACTGGTC
28	*CATTCGCAGGGTAACGGCCA*TGGCGCGAGTCAGACAC
29	TCGCACCATCTACGACAACC
30	CTACGAAGCTGACGCCGAAG
31	GTGGTGCTCGAGAACATAAG
32	CGGTCACCCGTAACAATCAG
33	CAGGATCTTATGCACATAGGACTG
dxs_E	CTGCGGCGTATTCAGAGTTC
Pa_*crtY*-fw	*CTGCAGGTCGACTCTAGAG***GAAAGGAGGCCCTTCAG**ATGCAACCGCATTATGATCTG
Pa_crtY-rv1	CGGTACCCGGGGATCTTAACGATGAGTCGTCATAATGG
Pa_crtY-rv2	GGCATTCCAAATCCACAACATCTGAAGGGCCTCCTTTCTTAACGATGAGTCGTCATAATGG
Pa_crtZ-fw2	*CCATTATGACGACTCATCGTTAA***GAAAGGAGGCCCTTCAG**ATGTTGTGGATTTGGAATGCC
Pa_crtZ-rv	CGGTACCCGGGGATCTTACTTCCCGGATGCGG
crtW-fw 2	*CATCCGGGAAGTAAGATCCCC***GAAAGGAGGCCCTTCAG**ATGACCGCCGCCGTCG
crtW-rv	*CGGTACCCGGGGATC*TCAAGACTCGCCGCGCCAC
A1	*CTGCAGGTCGACTCTAGAG***GAAAGGAGGCCCTTCAG**ATGACCGCCGCCGTCG
A2	*CGGTACCCGGGGATC*TCAAGACTCGCCGCGCCAC
A3	*CAGATCATAATGCGGTTGCAT***CTGAAGGGCCTCCTTTC**TCAAGACTCGCCGCGCCAC
A4	*GTGGCGCGGCGAGTCTTGA***GAAAGGAGGCCCTTCAG**ATGCAACCGCATTATGATCTG
A6	*CTGCAGGTCGACTCTAGAG***GAAAGGAGGCCCTTCAG**ATGTTGTGGATTTGGAATGCC
A7	*CGACGGCGGCGGTCAT***CTGAAGGGCCTCCTTTC**TTACTTCCCGGATGCGG
A8	*CCGCATCCGGGAAGTAA***GAAAGGAGGCCCTTCAG**ATGACCGCCGCCGTCG
pVWEx-fw	CATCATAACGGTTCTGGC
pVWEx-rv	ATCTTCTCTCATCCGCCA
M13 fw	CACAGCGGGAGTGCCTATTGTTTTG
M13 rv	CAGCGATGATCACTTCTGGCTC

*Sequence in bold: artificial ribosome binding site; sequence underlined: restriction site; sequence in italics: linker sequence for hybridization*.

### Deletion of carotenogenic genes in *C. glutamicum* MB001

For deletion of the carotenogenic genes *crtY_e/f_* and *crtEb*, encoding the C45/C50 carotenoid ε-cyclase and the lycopne elongase, respectively, the suicide vector pK19*mobsacB* was used (Schäfer et al., 1994). Genomic regions flanking the *crtYEb* cluster were amplified from genomic DNA of *C. glutamicum* WT using primer pairs *crtY*-A/*crtY*-B and *crtEb*-C/*crtEb*-D (Table 2), respectively. The PCR products were purified and linked by crossover PCR using the primer pair *crtY*-A/*crtEb-*D (Table 2). The purified PCR product was cloned into pK19*mobsacB* resulting in the construction of deletion vector pK19*mobsacB*-δ*crtYEb* (Table 1). The targeted deletion of *crtYEb* via two-step homologous recombination as well as the selection for the first and second recombination events were carried out as described previously (Eggeling and Bott, 2005). Deletion of *crtYEb* was verified by PCR analysis of the constructed mutant using primer pair *crtY-*E/*crtEb*-F (Table 2).

### Construct design of the synthetic MEP operons and their integration into the genome of *C. glutamicum* LYC3

The integration of the synthetic operons Op1 and Op2 was conducted by using the suicide vector pK19*mobsacB* (Schäfer et al., 1994). Op1 consists of the MEP-pathway genes *ispD*, *ispF*, and *ispE* under the control of the constitutive P*_tuf_* promoter. *IspD* and *ispF* form a transcription unit and were amplified as such from genomic DNA from *C. glutamicum* WT using the oligonucleotides 5 and 6. The primer pair 7/8 was used to amplify *ispE* from *C. glutamicum* WT, introducing an artificial ribosome binding site (RBS) in front of the gene. The promoter region was amplified using the oligonucleotides 3 and 4. In Op2 *dxr*, *ispG* and *ispH* were combined, by amplification from the *C. glutamicum* WT genome using the primer pairs 15/16, 17/18, and 19/20, respectively. An artificial RBS in front of *ispG* and *ispH* each was introduced by the oligonucleotides 17 and 19, respectively. Also the genes of Op2 were put under the control of the P*_tuf_* promoter, amplified from genomic DNA using the primers 13 and 14. Genomic regions flanking the selected insertion region were amplified from genomic DNA of *C. glutamicum* LYC3 using primer pairs 1/2 and 9/10 for integration in the cgp2 cured region in the case of Op1, or 11/12 and 20/22 for integration of Op2 in the cgp1 cured region (Table 2), respectively. The purified PCR products were either linked by crossover PCR or were directly combined together with the plasmid by Gibson assembly (Gibson et al., 2009). The final assembly of the insert with linearized pK19*mobsacB* led to the construction of the respective integration vectors pK19*mobsacB*-Op1 and pK19*mobsacB*-Op2 (Table 1). The following integration of the operon by two-step homologous recombination was performed according to the deletion of genes. The integration of operon1 and 2 was verified by PCR using the primers 29/30 and 31/32, respectively.

### Promoter exchange of the *dxs* gene in *C. glutamicum* LYC3

The plasmid pK19*mobsacB-*P*_tuf_dxs* was constructed to replace the native *dxs* promoter with the *tuf* promoter region from *C. glutamicum* WT. For this purpose, the upstream region of *dxs* (483 bp), the 3′ part of *dxs* and the *tuf* promoter region [200 bp upstream of the coding sequence of the *tuf* gene(cg0587)] were amplified from chromosomal DNA of *C. glutamicum* LYC3 using the oligonucleotide pairs 27/28, 23/24, and 25/26, respectively (Table 2). By crossover PCR, the *dxs* 3′ fragment and the *tuf* promoter region were fused with oligonucleotides 23/26. Afterward, the *dxs* upstream region was fused to this 644 bp long fragment using oligonucleotides 27/26. The final purified PCR product was cloned into pK19*mobsacB* resulting in the vector pK19*mobsacB-*P*_tuf_dxs* (Table 1). The following process for the promoter exchange by two-step homologous recombination was performed as described earlier for the deletion of genes. The promoter exchange was verified by PCR using the primers dxs_E and 33, and sequencing of the PCR product.

### Overexpression of carotenogenic genes

Plasmids harboring a carotenogenic gene (general abbreviation *crt*), pEKEx3-*crt* or pVWEx1-*crt* allowed an IPTG-inducible overexpression of *crt*. They were constructed on the basis of pEKEx3 (Stansen et al., 2005) or pVWEx1 (Peters-Wendisch et al., 2001), respectively. Amplification of *crt* by polymerase chain reaction (PCR) from genomic DNA of *C. glutamicum* WT, *P. ananatis* and *B. aurentiaca*, which was prepared as described (Eikmanns et al., 1995), was carried out using the respective primers (Table 2). The amplification of the *crt* genes from was based on genomic DNA as template. The amplified products were cloned into the appropriately restricted pEKEx3 or pVWEx1 plasmid DNA.

### Extraction analysis of carotenoids

To extract carotenoids from the *C. glutamicum* strains 15 ml aliquots of the cell cultures were centrifuged at 10,000 × g for 15 min and the pellets were washed with deionized H_2_O. The pigments were extracted with 10 ml methanol:acetone mixture (7:3) at 60°C for 30 min with thorough vortexing every 10 min. When necessary, several extraction cycles were performed to remove all visible colors from the cell pellet (Heider et al., 2012).

The extraction mixture was centrifuged 10,000 × g for 15 min and the supernatant was transferred to a new tube. The carotenoid content in the extracts was quantified through absorbance at 470 nm by HPLC analysis (see below) and the concentrations were calculated using a standard curve and appropriate dilutions. High performance liquid chromatography (HPLC) analyses of the *C. glutamicum* extracts were performed like described earlier (Heider et al., 2014a) on an Agilent 1200 series HPLC system (Agilent Technologies Sales & Services GmbH & Co., KG, Waldbronn), including a diode array detector (DAD) for UV/visible (Vis) spectrum recording. For separation, a column system consisting of a precolumn (10 mm × 4 mm MultoHigh 100 RP18-5, CS Chromatographie Service GmbH, Langerwehe, Germany) and a main column (ProntoSIL 200-5 C30, 250 mm × 4 mm, CS Chromatographie Service GmbH, Langerwehe, Germany) was used. Quantification of carotenoids was performed using the extracted wavelength chromatogram at 470 nm for decaprenoxanthin and carotenoids with corresponding UV/Vis profiles as well as for lycopene and corresponding carotenoids. Lycopene from tomato (Sigma, Steinheim, Germany), astaxanthin (Ehrenstorfer GmbH, Augsburg, Germany), and β-carotene (Merck, Darmstadt, Germany) were used as standards. The carotenoids were dissolved in chloroform according to its solubility and diluted in methanol:acetone (7:3). Due to the lack of appropriate standards decaprenoxanthin and zeaxanthin quantification was calculated based on a β-carotene standard and reported as β-carotene equivalents. The HPLC protocol comprised a gradient elution for 10 min and a mobile phase composition of (A) methanol and (B) methanol/methyl tert-butyl ether/ethyl acetate (5:4:1) starting from 10 to 100% eluent B followed by 20 min of isocratic elution with 100% B. After that, the eluent composition is set back to 10% B for 3 min. The injection volume was 50 μl and the flow rate was kept constant at 1.4 ml/min.

### DXS activity assay

The DXS activity of *C. glutamicum* crude extracts was determined using an endpoint assay adopted from Xiang et al. (2007), which is based on the measurement of the remaining pyruvate level in the reaction mixture. The assays were carried out at 30°C in total volume of 1 ml containing 50 mM Tris (pH 7.5), 60 μM pyruvate, 60 μM GAP, 10 mM dithiothreitol (DTT), 5 mM MgCl_2_, and 600 μM TPP. Reactions were stopped after 5, 15, 30, and +60 min of incubation by heat inactivation (5 min at 95°C). Subsequent the leftover pyruvate was converted to lactate with lactate dehydrogenase and the concomitant consumption of NADH was determined by fluorescence. Therefore, the reaction was allowed to proceed for 60 min at room temperature. Then, 2.5 U ml^−1^ lactate dehydrogenase and 0.1 mM NADH was added to the reaction mixture and incubated for 30 min at 37°C. The NADH diminution was determined photometrically at 340 nm.

## Results

### Overexpression of *dxs* increased lycopene yield

The first and often rate-limiting reaction in the MEP pathway is the condensation of pyruvate and GAP to DXP catalyzed by Dxs (Harker and Bramley, 1999; Kim and Keasling, 2001). To test if Dxs is a bottleneck in carotenoid biosynthesis in *C. glutamicum*, *dxs* was overexpressed in *C. glutamicum* LYC3, a mutant derived from the genome-reduced *C. glutamicum* strain MB001 (Baumgart et al., 2013) that accumulates lycopene due to deletion of the lycopene elongase and C45/C50 carotenoid ε-cyclase genes *crtEb* and *crtY_e/f_*. To exchange the native *dxs* promoter by the strong constitutive promoter of *tuf* (cg0587), which encodes for the elongation factor EF-Tu (Fukui et al., 2011), the replacement vector pK19mobsacB-P_tuf_*dxs* was constructed and *C. glutamicum* LYC3-P_tuf_*dxs* was obtained. Dxs activities measured in crude extracts were about twofold higher in *C. glutamicum* LYC3-P_*tuf*_*dxs* (16 ± 1 mU mg^−1^) than in the control strain *C. glutamicum* LYC3 (Table 3). As consequence of enhanced Dxs activity, lycopene production doubled (0.08 ± 0.01 mg g^−1^ DCW as compared to 0.04 ± 0.01 mg g^−1^ DCW) (Table 3). Thus, increased Dxs activity improved lycopene production by *C. glutamicum*. Increased specific Dxs activities were also observed when a plasmid-borne copy of *dxs* was overexpressed from an IPTG-inducible promoter in LYC3, but lycopene production was only slightly improved (Table 3). Hence, chromosomal overexpression proved better and was therefore chosen for subsequent metabolic engineering of the MEP pathway.

**Table 3:** **Influence of chromosomal promoter exchange of the 1-deoxy-d-xylulose 5-phosphate synthase gene *dxs* on Dxs actitivities, growth rates, and lycopene production**.

*C. glutamicum* strain	Growth rate (h^−1^)	final OD (600 nm)	Dxs sp. act. (mU mg^−1^)	Lycopene production (mg g^−1^ DCW)
LYC3	0.45 ± 0.01	27 ± 1	9 ± 1	0.04 ± 0.01
LYC3-P_*tuf*_*dxs*	0.44 ± 0.02	24 ± 2	16 ± 1	0.08 ± 0.01
LYC3(pEKEx3-dxs)	0.38 ± 0.01	22 ± 2	26 ± 3	0.06 ± 0.01

*Cells were grown in glucose CGXII minimal medium for 24 h. Means and standard deviations of three cultivations are reported*.

### Overproduction of enzymes converting DXP to IPP using two synthetic operons integrated into the *C. glutamicum* chromosome

**Figure 2:**
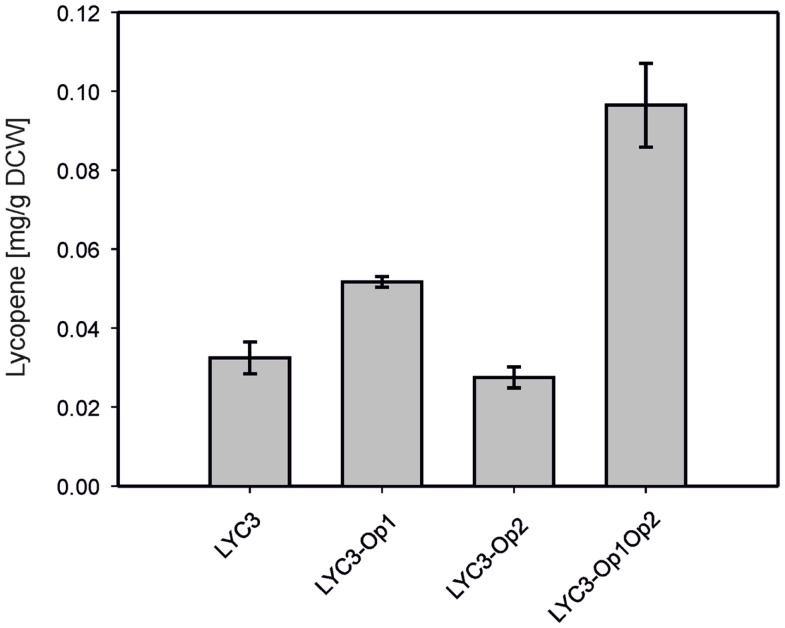
**Lycopene production by *C. gluamicum* LYC3 and derived strains expressing the synthetic *ispDFE* operon (LYC3-Op1), the synthetic *dxr-ispGH* operon (LYC3-Op2) or both operons (LYC3-Op1Op2) for overproduction of MEP pathway enzymes**. Cells were grown in glucose CGXII minimal medium. Means and standard deviations of three cultivations are shown.

For overproduction of the six MEP pathway enzymes catalyzing the conversion of DXP to IPP, two synthetic operons were constructed and integrated into the chromosome of *C. glutamicum* LYC3. Operon 1 was constructed to drive expression of *ispDF*, which are cotranscribed naturally, fused to *ispE* from P*_tuf_*. The RBS of the *tuf* gene was inserted upstream of *ispD*, while the endogenous RBS of *ispF* and a perfect *C. glutamicum* RBS upstream of *ispE* were used. To construct operon 2, *dxr*, *ispG*, and *ispH* were fused for expression from P*_tuf_* and perfect *C. glutamicum* RBS were inserted upstream of *ispG* and *ispH* while the RBS of the *tuf* gene was used upstream of *dxr*. Both operons were integrated by homologous recombination into the chromosome of *C. glutamicum* LYC3, which lacks prophages cgp1 and cgp2. Operon 1 was integrated into the chromosome of *C. glutamicum* LYC3 between cg1506 and cg1525, i.e., at the position that harbors prophage cgp2 in the *C. glutamicum* WT, but which is absent from LYC3, and the resulting strain was named LYC3-Op1. Similarly, *C. glutamicum* LYC3-Op2 was obtained by integrating operon 2 into the chromosome of *C. glutamicum* LYC3 at the position (between cg1745 and cg1753) that in *C. glutamicum* WT harbors prophage cgp1, but which is absent from LYC3. The constructed *C. glutamicum* strain LYC3-Op1Op2 contains both operons in the chromosome instead of prophages cgp1 and cgp2. *C. glutamicum* LYC3-Op1 showed slightly higher lycopene accumulation than *C. glutamicum* strains LYC3 and LYC3-Op2. *C. glutamicum* LYC3-Op2 grew slower than LYC3 and LYC3-Op1. *C. glutamicum* LYC3-Op1Op2 that harbors both operons also grew slower, but accumulated almost threefold more lycopene than LYC3. Thus, overexpression of MEP pathway genes from two chromosomally integrated synthetic operons improved lycopene production (Figure 2).

### Improved IPP supply by chromosome-based enhancement of MEP pathway gene expression

**Figure 3:**
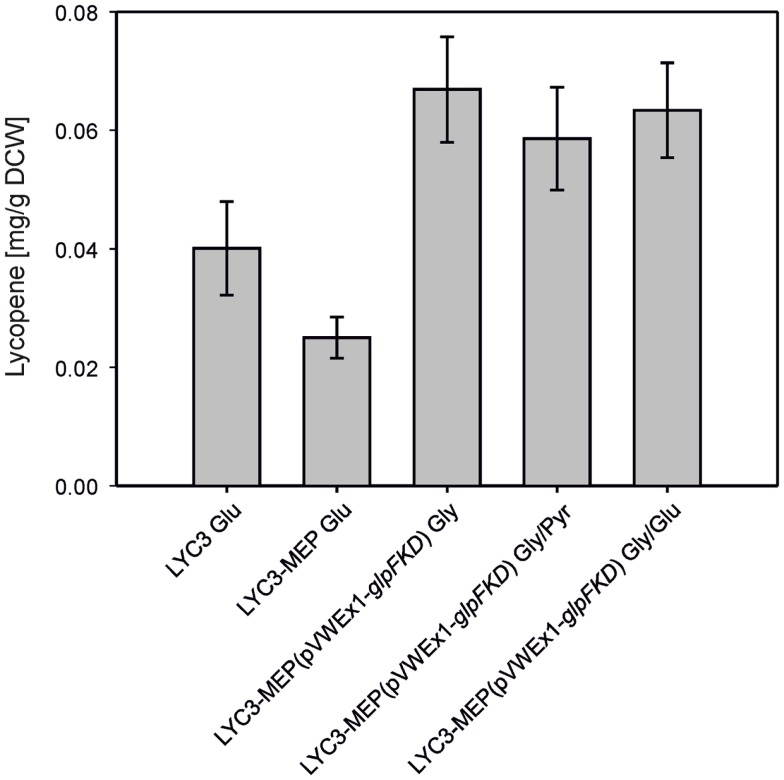
**Lyopene production by *C. gluamicum* LYC3-MEP(pVWEx1- *glpFKD*) on glycerol as sole and combined carbon source**. LYC3-MEP (pVWEx1-*glpFKD*) cells were grown in CGXII minimal medium with 200 mM glycerol (Gly), 100 mM glycerol + 100 mM pyruvate (Gly/Pyr), or 100 mM glycerol + 50 mM glucose (Gly/Glu), respectively. Expression of *glpFKD* was induced by 50 μM IPTG. As reference, lycopene production of the strains LYC3 and LYC3-MEP grown in CGXII minimal medium with 100 mM glucose (Glu) is given. Means and standard deviations of three cultivations are reported.

To combine chromosome-based overexpression of the genes necessary for conversion of DXP to IPP with overproduction of Dxs, the first enzyme of the MEP pathway, the endogenous promoter of chromosomal *dxs* was exchanged by P*_tuf_* in *C. glutamicum* LYC3-Op1Op2 and the resulting strain was named *C. glutamicum* LYC3-MEP. Surprisingly, LYC3-MEP showed slower growth on solid as well as in liquid medium. Poor growth in liquid glucose medium was accompanied by little lycopene production, although LYC3-MEP colonies appeared well pigmented on plates. Since the central carbon metabolites pyruvate and GAP are the immediate precursors of the MEP pathway, it was tested if lycopene production by *C. glutamicum* LYC3-MEP was affected by the carbon source. To this end, pyruvate and glycerol were tested as carbon sources. Since glycerol is no carbon source for *C. glutamicum* WT, *glpFKD* from *E. coli* encoding the enzyme for conversion of glycerol to GAP were expressed from plasmid pVWEx1-*glpFKD* (Rittmann et al., 2008) in *C. glutamicum* LYC3-MEP. Growth by *C. glutamicum* LYC3-MEP(pVWEx1-*glpFKD*) on glycerol, glycerol + glucose, or glycerol + pyruvate was still impaired, but about twofold more lycopene (around 0.07 ± 0.01 mg g^−1^ DCW) accumulated than with glucose as sole carbon source (Figure 3).

Since IspH synthesizes both IPP and DMAPP, but typically not in equimolar amounts (Rohdich et al., 2002; Gräwert et al., 2004; Xiao et al., 2008), it is possible that unbalanced biosynthesis of IPP and DMAPP in *C. glutamicum* LYC3-MEP impairs growth and carotenogenesis. To test this hypothesis, isopentenyl pyrophosphate isomerase Idi was overproduced. Indeed, *C. glutamicum* LYC3-MEP(pVWEx1-idi)(pEKEx3) produced twofold more lycopene (0.08 ± 0.02 mg g^−1^ DCW) than *C. glutamicum* strains LYC3, LYC3-MEP, and the empty vector control strain, but still showed impaired growth (Table 4). Thus, a lycopene producing *C. glutamicum* strain with improved IPP supply overexpressing all MEP pathway genes and *idi* could be constructed. However, lycopene production by this strain (Table 4) was comparable to that by *C. glutamicum* strains LYC3-P_tuf_*dxs* (Table 3) and LYC3-Op1Op2 (Figure 2) indicating that the positive effects did not act synergistically. This was also observed when the strains were grown in LB medium supplemented with 100 mM glucose; however, they grew faster (data not shown). Taken together, *C. glutamicum* strains with improved IPP and DMAPP supply showed higher lycopene production than the respective parental strains.

**Table 4:** **Growth rates and lycopene production by prophage-cured, MEP pathway genes overexpressing *C. glutamicum* strain LYC3-MEP**.

*C. glutamicum* strain	Growth rate (h^−1^)	final OD (600 nm)	Lycopene production (mg g^−1^ DCW)
LYC3	0.45 ± 0.01	27 ± 1	0.04 ± 0.01
LYC3-MEP	0.16 ± 0.01	23 ± 1	0.03 ± 0.01
LYC3-MEP(pVWEx1)(pEKEx3)	0.15 ± 0.00	23 ± 2	0.04 ± 0.02
LYC3-MEP(pVWEx1-*idi*)(pEKEx3)	0.13 ± 0.01	20 ± 1	0.08 ± 0.02

*Cells were grown in glucose CGXII minimal medium and plasmid carrying strains were induced with 50 μM IPTG. Means and standard deviations of three cultivations are shown*.

### Application of *C. glutamicum* with improved IPP supply for production of decaprenoxanthin and astaxanthin

To test if *C. glutamicum* LYC3-MEP overexpressing *idi* is suitable for production of the endogenous C50 carotenoid decaprenoxanthin, this strain was transformed with plasmid pEKEx3-*crtEbY*. Expression of lycopene elongase gene *crtEb* and of carotenoid ε-cyclase gene *crtY_e/f_* from this plasmid complements the lycopene producing *C. glutamicum* LYC3-MEP, which carries chromosomal *crtEb* and *crtY_e/f_* deletions allowing for decaprenoxanthin biosynthesis. The resulting strain LYC3-MEP(pVWEX1-*idi*)(pEKEx3-*crtEbY*) overproduces all enzymes of endogenous carotenogenesis except *crtE*, *crtB*, and *crtI* (Figure 1). Although it grew slowly, LYC3-MEP (pVWEX1-*idi*)(pEKEx3-*crtEbY*) produced 0.35 ± 0.02 mg g^−1^ DCW (Table 5) and, thus, is a genome-reduced strain with improved IPP supply suitable for the overproduction of the endogenous C50 carotenoid decaprenoxanthin.

**Table 5:** **Astaxanthin and decaprenoxanthin production by recombinant *C. glutamicum* strains with improved IPP supply**.

*C. glutamicum* strain	Production (mg g^−1^ DCW)			
	Decaprenoxanthin	β-Carotene	Zeaxanthin	Astaxanthin
LYC3-MEP(pVWEx1)(pEKEx3)	<0.01	<0.01	<0.01	<0.01
LYC3-MEP(pVWEx1-*idi*)(pEKEx3-*crtEbY*)	0.4 ± 0.1	<0.01	<0.01	<0.01
LYC3-MEP(pVWEx1-*idi*)(pEKEx3-*crtZWY*)	<0.01	<0.01	<0.01	0.1 ± 0.0
LYC3-MEP(pVWEx1-*crtEBI*)(pEKEx3-*crtZWY*)	<0.01	2.1 ± 1.3	1.2 ± 0.2	1.2 ± 0.5

*Cells were grown in glucose CGXII minimal medium with 50 μM IPTG. Means and standard deviations of three cultivations are reported*.

*C. glutamicum* has previously been engineered for the production of the non-native C40 carotenoids β-carotene and zeaxanthin (Heider et al., 2014a). When *crtY*_Pa_ (PANA_4160) encoding lycopene cyclase from *Pantoea ananatis* was expressed, β-carotene accumulated. Additional expression of *crtZ*_Pa_ (PANA_4163), which encodes β-carotene hydroxylase, resulted in partial conversion of β-carotene to zeaxanthin (Heider et al., 2014a). To enable astaxanthin production, *crtW*_Ba_ encoding carotene C(4) oxygenase from *Brevundimonas aurantiaca*, which oxidizes zeaxanthin to yield astaxanthin, was expressed in addition to *crtY*_Pa_ and *crtZ*_Pa_. The resulting plasmid pEKEx3-*crtZWY* was used to transform LYC3-MEP(pVWEX1-*idi*). *C. glutamicum* LYC3-MEP(pVWEX1-*idi*)(pEKEx3-*crtZWY*) produced 0.14 ± 0.01 mg g^−1^ DCW astaxanthin and neither β-carotene nor zeaxanthin accumulated (Table 5). Thus, to the best of our knowledge, this is the documentation of astaxanthin production by recombinant *C. glutamicum*. Although levels were low, LYC3-MEP(pVWEX1-*idi*)(pEKEx3-*crtZWY*) produced astaxanthin as only carotenoid.

Based on our previous findings that overexpression of the genes *crtE*, *crtB*, and *crtI* (Figure 1) strongly increased lycopene production (Heider et al., 2012), as well as decaprenoxanthin production (Heider et al., 2014a); these genes were overexpressed from plasmid pVWEx3-*crtEBI*. The resulting strain *C. glutamicum* LYC3-MEP(pVWEx3-*crtEBI*)(pEKEx3-*crtZWY*) produced 2.1 ± 1.3 mg g^−1^ DCW β-carotene and 1.2 ± 0.2 mg g^−1^ DCW zeaxanthin (Table 5), but also ninefold more astaxanthin (1.2 ± 0.5 mg g^−1^ DCW) than LYC3-MEP(pVWEx1-*idi*)(pEKEx3-*crtZWY*). Thus, it was shown that astaxanthin can be produced by recombinant *C. glutamicum* in the milligrams per gram DCW range.

## Discussion

Recently, *C. glutamicum* has been engineered for production of diverse lycopene-derived carotenoids (Heider et al., 2014a) and of a sesquiterpene (Frohwitter et al., 2014). There is an increasing demand for efficient, low-cost, and natural production of terpenoids (Zhu et al., 2014) as they have many applications, e.g., in the medicinal and nutraceutical industries or as fuels (Martin et al., 2003; Ajikumar et al., 2010; Peralta-Yahya et al., 2011). Besides terminal terpenoid pathway engineering, an efficient supply of the prenyl pyrophosphate precursors is important (Heider et al., 2014b). It could be shown here that MEP pathway engineering to improve IPP supply in *C. glutamicum* improved lycopene production. However, as observed in similar studies of MEP pathway engineering in other bacteria individual bottlenecks may be overcome, but the individual beneficial effects do not necessarily add up (Kim and Keasling, 2001; Martin et al., 2003; Rodriguez-Villalon et al., 2008). Overexpressing the initial MEP pathway gene, *dxs* improved lycopene production by *C. glutamicum* (see Figure 1) and by other bacteria (Harker and Bramley, 1999; Matthews and Wurtzel, 2000). However, optimal overexpression levels need to be established since, e.g., chromosomal overexpression proved better than overexpression from a multy-copy plasmid (Yuan et al., 2006). Similarly, when *dxs* was overexpressed in *C. glutamicum* by exchanging the native promoter of *dxs* with the strong constitutive *tuf* promoter more lycopene accumulated than when plasmid-borne *dxs* overexpression, which led to higher Dxs activities, was tested (Table 3). The complex interplay of MEP pathway enzymes is also reflected by the fact that overexpression of *dxr*, *ispG*, and *ispH* in LYC3-Op2 only improved lycopene accumulation when combined with overexpression of *ispDF* and *ispE* (Op1) (Figure 2). Although lycopene titers obtained with *C. glutamicum* LYC3-Op1Op2 were comparable to the *dxs* overexpressing strain LYC3-P*_tuf_dxs* (Figure 2 and Table 3), their combination in strain LYC3-MEP was not synergistic and even perturbed growth. This may be explained by accumulation of inhibitory MEP pathway intermediates as shown for *B. subtilis* (Sivy et al., 2011) and *E. coli* (Martin et al., 2003; Zou et al., 2013), from an excessive drain of central metabolic intermediates (Kim and Keasling, 2001) and/or from an imbalance between IPP and DMAPP (Kajiwara et al., 1997). In *C. glutamicum*, improved lycopene production as consequence of overexpression of IPP isomerase gene *idi* was observed in LYC3-MEP (Table 4). However, lycopene production by LYC3-MEP overexpressing *idi* was not higher than by LYC3-P*_tuf_dxs* or by LYC3-Op1Op2. Moreover, when *dxs* was overexpressed in the WT-derived strain Δ*crtEb* lycopene production increased from about 0.04 to about 0.12 mg g^−1^ DCW, but combined overexpression of *dxs* and *idi* did not further increase lycopene production (data not shown). Thus, the perturbed growth may not only be due to an imbalance between IPP and DMAPP.

It remains to be shown if combinatorial approaches to optimize multiple gene expression levels (Zelcbuch et al., 2013; Nowroozi et al., 2014) would improve the IPP precursor supply in *C. glutamicum*. Fine-tuning of gene expression in recombinant *C. glutamicum* by varying promoters (Holátko et al., 2009; van Ooyen et al., 2011; Schneider et al., 2012), RBSs (Schneider et al., 2012), translational start codons (Schneider et al., 2012), or translational stop codons (Jensen and Wendisch, 2013) improved production of amino acids and diamines. In addition, overexpression of heterologous instead of endogenous genes may be beneficial, e.g., as shown for improving isoprene production by *E. coli* via overexpression of two MEP pathway genes *dxs* and *dxr* from *B. subtilis* (Zhao et al., 2011) or by combining overexpression of *xylA* from *Xanthomonas campestris* with endogenous *xylB* to accelerate xylose utilization of *C. glutamicum* (Meiswinkel et al., 2013).

Besides fine-tuning of MEP pathway gene overexpression, growth, and terpenoid production by recombinant *C. glutamicum* with increased IPP supply could be improved by metabolic pull, i.e., by overexpression of genes of the downstream terpenoid pathway (Table 5). Similarly, amorphadiene synthase overexpression prevented accumulation of inhibitory isoprenoid pathway intermediates in *E. coli* (Martin et al., 2003). Overcoming the toxicity of accumulating IPP and DMAPP was successfully used as screening method for the identification of genes that are involved in isoprenoid biosynthesis (Withers et al., 2007). Accumulation of the MEP pathway intermediate ME-cPP inhibits growth and isoprenoid production by recombinant *E. coli*. To abolish its accumulation overexproducing the two enzymes downstream of ME-cPP (*ispG* and *ispH*) needed to be combined with overexpressing an operon for iron–sulfur cluster assembly since both IspG and IspH are containing iron–sulfur clusters (Zou et al., 2013).

To the best of our knowledge, production of astaxanthin by recombinant *C. glutamicum* was shown here for the first time. Astaxanthin is the third most important carotenoid after β-carotene and lutein and its global market amounted to about 230 million US$ in 2010 (BBC Research, 2011). The economically most significant application of astaxanthin is its use as feed additive in aquaculture industry (Lorenz and Cysewski, 2000; Higuera-Ciapara et al., 2006; Schmidt et al., 2011), but it also exhibits high potential as a nutraceutical and as an approved ingredient for cosmetics due to its remarkably high antioxidative activity (Miki, 1991; Schmidt et al., 2011). Astaxanthin is mainly produced by marine bacteria and microalgae, but only the green freshwater microalga *Haematococcus pluvialis* and the red yeasts *Xanthophyllomyces dendrohous/Phaffia rhodozyma* are established as hosts for commercial production (Bhosale and Bernstein, 2005; Rodriguez-Saiz et al., 2010). Algae-based production of astaxanthin is still more costly than chemical synthesis (Jackson et al., 2008), but markets more and more demand naturally produced carotenoids. The astaxanthin titers by recombinant *C. glutamicum* reported here are in the milligrams per gram DCW range and, thus, they are comparable to yields described for *P. rhodozyma* (ranging from 0.16 to 6.6 mg g^−1^ DCW (Cruz and Parajo, 1998; Jacobson et al., 1999). The highest product titer of 9.7 mg g^−1^ DCW is reported for a *P. rhodozyma* strain improved by metabolic engineering and classical mutagenesis (Gassel et al., 2013), while the highest titer in a recombinant bacterium, i.e., *E. coli* strain was 5.8 mg g^−1^ DCW astaxanthin (Zelcbuch et al., 2013). Thus, the astaxanthin titers reported for *C. glutamicum* are comparable and it is conceivable that they may be improved further by combining metabolic engineering with classical mutagenesis as in *P. rhodozyma* (Gassel et al., 2013), by combinatorial approaches to gene expression (Zelcbuch et al., 2013), or by high-cell density cultivation since biomass concentrations of up to 95 g DCW/l have been reported for *C. glutamicum* (Riesenberg and Guthke, 1999).

## Conflict of Interest Statement

The authors declare that the research was conducted in the absence of any commercial or financial relationships that could be construed as a potential conflict of interest.
